# Prognostic Value of Myocardial Blush Grade in Patients With ST-Segment Elevation Myocardial Infarction (STEMI) Undergoing Primary Percutaneous Coronary Intervention (PCI): Association With Reperfusion Success and In-Hospital Outcomes

**DOI:** 10.7759/cureus.96717

**Published:** 2025-11-12

**Authors:** Sher Wali Khan, Muhammad Ahtesham, Fazal E Azim, Muhammad Sajid, Ayesha Fayyaz, Ikram Ullah, Zarak Khan

**Affiliations:** 1 Adult Cardiology, Lady Reading Hospital Medical Teaching Institution, Peshawar, PAK; 2 Cardiology, Lady Reading Hospital Medical Teaching Institution, Peshawar, PAK; 3 Internal Medicine, State University of New York (SUNY) Downstate Medical Center, SUNY Downstate Health Sciences University, Brooklyn, USA; 4 Cardiology, Northwest General Hospital and Research Centre, Peshawar, PAK; 5 Cardiology, Peshawar Institute of Cardiology Medical Teaching Institution, Peshawar, PAK

**Keywords:** in-hospital mortality, mbg, myocardial blush grade, primary pci, reperfusion success, stemi, st-segment elevation myocardial infarction, timi

## Abstract

Background: Myocardial blush grade (MBG) is a simple angiographic tool for assessing microvascular perfusion in patients with ST-segment elevation myocardial infarction (STEMI). While restoration of epicardial flow by primary percutaneous coronary intervention (PCI) is critical, microvascular reperfusion determines true myocardial salvage. Limited data are available from Pakistan regarding the prognostic value of MBG in predicting in-hospital outcomes.

Objective: This study aimed to evaluate the prognostic significance of MBG in STEMI patients undergoing primary PCI, with emphasis on reperfusion success and in-hospital adverse events.

Methods: This prospective observational study was conducted at Lady Reading Hospital Medical Teaching Institution, Peshawar, Pakistan, from January to December 2024. A total of 200 consecutive STEMI patients treated with primary PCI were included. MBG was assessed by two independent interventional cardiologists, and patients were stratified into low MBG (0-1) and high MBG (2-3) groups. Baseline demographics, angiographic findings, and in-hospital outcomes were compared using appropriate statistical tests. Multivariate logistic regression was applied to adjust for confounders.

Results: Among 200 patients, 80 (40%) had MBG 0-1 and 120 (60%) had MBG 2-3. Patients with low MBG were older, were more likely to present with anterior wall myocardial infarction* *(MI), and had longer door-to-balloon times. Post-PCI Thrombolysis in Myocardial Infarction grade 3 (TIMI 3) flow was achieved in 72.5% of MBG 0-1 versus 96.7% of MBG 2-3 patients (p<0.001). In-hospital mortality was significantly higher in the low MBG group (8.8% vs. 1.7%; OR: 5.58; 95% CI: 1.09-28.4; p=0.037). Low MBG was also associated with recurrent ischemia, acute heart failure, and reduced reperfusion success. On multivariate analysis, MBG 0-1 independently predicted in-hospital adverse outcomes (adjusted OR: 3.42; 95% CI: 1.48-7.91; p=0.004), along with prolonged door-to-balloon time and anterior wall MI.

Conclusion: MBG is a powerful and independent predictor of reperfusion success and in-hospital outcomes in STEMI patients undergoing primary PCI. Its routine use in clinical practice can aid in early risk stratification, especially in resource-limited settings such as Pakistan. Future multicenter studies with long-term follow-up are warranted to confirm these findings.

## Introduction

ST-segment elevation myocardial infarction (STEMI) continues to be a predominant cause of morbidity and mortality globally. Primary percutaneous coronary intervention (PCI) is the benchmark for reperfusion therapy, since it reinstates epicardial coronary artery flow and diminishes both short- and long-term unfavorable outcomes [1,2]. Nonetheless, angiographic success, as assessed by Thrombolysis in Myocardial Infarction (TIMI) flow grade, does not consistently indicate cardiac tissue-level reperfusion, and patients may persist in experiencing adverse clinical outcomes despite attaining TIMI 3 flow [3,4].

The myocardial blush grade (MBG) has become a straightforward, reproducible angiographic indicator of microvascular reperfusion. It visually evaluates myocardial perfusion by analyzing the "blush" of contrast within the myocardium, thus offering an indirect assessment of capillary flow [5]. Prior research has shown that compromised MBG correlates with greater infarct size, diminished left ventricular ejection fraction, and elevated death rates [6,7]. Consequently, MBG provides additional prognostic insights beyond the restoration of epicardial flow.

Numerous international studies have confirmed the predictive value of MBG; however, its use in standard clinical practice is still restricted, particularly in low- and middle-income nations [5,6]. Cardiovascular disease is the predominant cause of mortality in Pakistan, with STEMI representing a significant portion of acute coronary syndromes [8,9].

Notwithstanding the proliferation of PCI facilities at tertiary care centers, obstacles such as delayed patient presentation, limited public awareness, and financial constraints continue to adversely influence clinical outcomes [10,11]. Consequently, evaluating angiographic indicators such as MBG in the local population may enhance post-PCI risk stratification and guide evidence-based therapeutic decisions.

Therefore, this study aimed to evaluate the prognostic significance of MBG in patients with STEMI undergoing primary PCI, specifically assessing its association with angiographic reperfusion success and short-term in-hospital adverse outcomes.

## Materials and methods

This prospective observational study was conducted in the Department of Cardiology at Lady Reading Hospital (LRH) Medical Teaching Institution, Peshawar, Pakistan, over the course of one year, from January 2024 to December 2024 after obtaining approval from the institute's Institutional Review Board (approval number: 963/LRH/MTI; date: January 5, 2024).

The study population comprised consecutive patients diagnosed with STEMI who underwent primary PCI as the initial reperfusion strategy. Patients were considered eligible if they were 18 years or older, presented within 12 hours of symptom onset, and received primary PCI. Patients with a history of coronary artery bypass graft (CABG) surgery, those presenting with cardiogenic shock upon admission, those who had received fibrinolytic therapy before PCI, and those with incomplete angiographic data were excluded from the study.

The sample size was determined using the World Health Organization (WHO) sample size calculator [12], applying a 95% confidence level and an 80% statistical power. Prior evidence suggested a 25% difference in reperfusion success between patients with higher MBG (2-3) and those with lower MBG (0-1). More recently, Che et al. [13] demonstrated that myocardial ischemia-reperfusion injury (MIRI) occurs frequently in STEMI patients undergoing PCI, with serum soluble suppression of tumorigenicity 2 (sST2) levels identified as an independent predictor of adverse outcomes. Patients with elevated serum sST2 concentrations (>68.98 ng/mL) were shown to have a markedly higher risk of developing MIRI and subsequent major adverse cardiovascular and cerebrovascular events (MACCEs), with an overall incidence of 17% and all-cause mortality of 3.6% during follow-up [13]. Based on these event rates, the minimum required sample size was calculated to be 180 cases. To allow for possible exclusions and incomplete data, the sample size was increased by 10%, yielding a final requirement of 200 patients. All consecutive eligible patients presenting during the study period were enrolled until the target sample size was achieved.

The diagnosis of STEMI was established according to standard clinical and electrocardiographic criteria [14,15]. These included chest pain lasting longer than 30 minutes and ST-segment elevation in at least two contiguous leads, defined as ≥1 mm in limb leads (or ≥0.1 mV in leads other than V2-V3 as per the European Society of Cardiology/American College of Cardiology Foundation/American Heart Association/World Heart Federation (ESC/ACCF/AHA/WHF) recommendations) or ≥2 mm in precordial leads.

Primary PCI was performed via either radial or femoral access, depending on the operator's preference and the patient's clinical condition. Standard interventional techniques such as guidewire passage, balloon dilation, and stent implantation were employed for the revascularization of the infarct-related artery. Coronary angiography was carried out to assess reperfusion quality. All interventional procedures were performed following standardized institutional protocols aligned with the European Society of Cardiology (ESC) guidelines to ensure procedural uniformity. Device selection, stent sizing, and contrast administration were standardized according to operator consensus protocols established prior to study initiation. Detailed procedural checklists were used to maintain consistency across all cases.

Following successful stent placement, both the TIMI flow grade [16] and MBG [17] were documented. MBG was graded visually on a scale from 0 to 3, where grade 0 indicated no myocardial blush, grade 1 denoted minimal blush, grade 2 represented moderate blush less than that of non-infarct-related arteries, and grade 3 corresponded to normal blush comparable to non-infarct-related arteries. To ensure consistency and minimize observer-related variability, two experienced interventional cardiologists, each with a minimum of five years' angiographic experience, independently assessed MBG while being blinded to patient clinical data and outcomes. Prior to study initiation, both observers underwent a calibration session using 20 representative angiograms to standardize MBG interpretation. In cases of discrepancy between independent readings, a final grade was assigned through joint review and consensus. Inter-observer agreement was calculated for internal validation and found to be satisfactory (κ=0.84), indicating strong reliability.

Reperfusion success was defined as the achievement of post-procedural TIMI 3 flow in the infarct-related artery in combination with MBG 2-3. After PCI, patients were admitted to the coronary care unit (CCU) and closely monitored throughout hospitalization. Clinical, angiographic, and outcome-related data were recorded in a structured database. The in-hospital outcomes of interest included recurrent ischemia, malignant arrhythmias, acute heart failure, cardiogenic shock, stroke, and in-hospital death.

Several measures were adopted to minimize potential sources of bias. Selection bias was reduced by enrolling all consecutive eligible patients during the study period. Information bias was minimized by employing consistent diagnostic criteria and standardized data collection tools. Observer bias in angiographic grading was reduced through blinded, independent assessments conducted by two interventional cardiologists. Confounding factors were addressed by applying multivariate regression models during the analysis. To facilitate reproducibility, all study procedures, including patient selection, data recording, and angiographic grading, were documented in a pre-specified protocol accessible to all investigators. Inter-observer reliability for MBG was reassessed midway through the study to ensure sustained consistency, with periodic cross-validation sessions conducted to prevent interpretive drift.

All data were analyzed using IBM SPSS Statistics for Windows, V. 27.0 (2020; IBM Corp., Armonk, NY, USA). Continuous variables were expressed as mean±standard deviation (SD) and compared between groups using the independent samples t-test. Categorical variables were summarized as frequencies and percentages and analyzed using the chi-squared (χ²) test or Fisher's exact test, as appropriate. Binary logistic regression analysis was performed to identify independent predictors of adverse in-hospital outcomes, with results presented as odds ratios (ORs) and 95% confidence intervals (CIs). A two-tailed p-value of <0.05 was considered statistically significant.

## Results

Table 1 shows the baseline clinical and demographic characteristics of the study population. Patients in the MBG 0-1 group were significantly older compared to those with MBG 2-3 (59.8±11.4 vs. 56.1±10.3 years; p=0.018). The prevalence of male sex, hypertension, diabetes, and smoking did not differ significantly between groups. However, anterior wall myocardial infarction (MI) was more frequent in the MBG 0-1 group (57.5% vs. 40.8%; p=0.024). The mean door-to-balloon time was also significantly longer among patients with low MBG (81.4±20.7 vs. 72.6±18.9 minutes; p=0.001).

**Table 1:** Baseline clinical and demographic characteristics of patients by MBG Patients with MBG 0-1 were significantly older, had a higher frequency of anterior wall MI, and had longer door-to-balloon times compared to MBG 2-3. Other baseline characteristics were not significantly different. Data are presented as mean±SD or n (%). MBG grading was performed according to van't Hof et al. [17] and STEMI diagnosis criteria per ESC/ACCF/AHA/WHF guidelines [14,15]. MBG: myocardial blush grade; MI: myocardial infarction; SD: standard deviation; CI: confidence interval; STEMI: ST-segment elevation myocardial infarction; ESC/ACCF/AHA/WHF: European Society of Cardiology/American College of Cardiology Foundation/American Heart Association/World Heart Federation

Variable	MBG 0-1 (n=80)	MBG 2-3 (n=120)	Statistical test	Test statistic	P-value	95% CI of difference
Age (years, mean±SD)	59.8±11.4	56.1±10.3	Independent t-test	t=2.39	0.018	0.65-6.65
Male sex, n (%)	61 (76.3%)	89 (74.2%)	χ² test	χ²=0.11	0.74	-
Hypertension, n (%)	48 (60%)	61 (50.8%)	χ² test	χ²=1.70	0.19	-
Diabetes mellitus, n (%)	32 (40%)	38 (31.7%)	χ² test	χ²=1.37	0.24	-
Current smoking, n (%)	36 (45%)	43 (35.8%)	χ² test	χ²=1.68	0.19	-
Anterior wall MI, n (%)	46 (57.5%)	49 (40.8%)	χ² test	χ²=5.06	0.024	-
Door-to-balloon time (min, mean±SD)	81.4±20.7	72.6±18.9	Independent t-test	t=3.39	0.001	3.6-13.9

The angiographic findings and procedural characteristics are summarized in Table 2. The prevalence of multivessel disease and the distribution of infarct-related arteries did not differ significantly between the groups. In both groups, the pre-PCI TIMI flow was comparable, ranging from 0 to 1. Nevertheless, patients with MBG 2-3 exhibited significantly higher rates of post-PCI TIMI 3 flow (96.7% vs. 72.5%; p<0.001), suggesting that those with higher MBG experienced superior epicardial reperfusion success.

**Table 2:** Angiographic findings and procedural characteristics Post-PCI TIMI 3 flow was significantly more frequent in MBG 2-3, indicating superior epicardial reperfusion. Other angiographic and procedural features were comparable between groups. Data are presented as n (%). TIMI flow grading was performed per Chesebro et al. [16] and MBG per van't Hof et al. [17]. PCI: percutaneous coronary intervention; TIMI: Thrombolysis in Myocardial Infarction; LAD: left anterior descending artery; MBG: myocardial blush grade; CI: confidence interval

Variable	MBG 0-1 (n=80)	MBG 2-3 (n=120)	Statistical test	Test statistic	P-value	95% CI of difference
Infarct-related artery: LAD	41 (51.3%)	56 (46.7%)	χ² test	χ²=0.35	0.55	-
Multivessel disease, n (%)	36 (45%)	39 (32.5%)	χ² test	χ²=3.05	0.08	-
Pre-PCI TIMI 0-1 flow, n (%)	71 (88.8%)	101 (84.2%)	χ² test	χ²=0.84	0.36	-
Post-PCI TIMI 3 flow, n (%)	58 (72.5%)	116 (96.7%)	χ² test	χ²=24.6	<0.001	-

Table 3 presents in-hospital outcomes. Reperfusion success was significantly greater in patients with MBG 2-3 (95.8% vs. 68.8%; p<0.001; OR: 8.85; 95% CI: 3.35-23.4). Conversely, MBG 0-1 patients experienced higher rates of recurrent ischemia (13.8% vs. 5%; p=0.031), acute heart failure (17.5% vs. 7.5%; p=0.027), and in-hospital mortality (8.8% vs. 1.7%; p=0.037). Although malignant arrhythmias and cardiogenic shock were more frequent in the low MBG group, the differences did not reach statistical significance.

**Table 3:** In-hospital clinical outcomes in patients by MBG group Reperfusion success was significantly higher in MBG 2-3, while MBG 0-1 was associated with increased rates of recurrent ischemia, acute heart failure, and mortality. Malignant arrhythmias and cardiogenic shock were more frequent in MBG 0-1, though not statistically significant. Data are presented as n (%). Reperfusion success was defined as TIMI 3 flow plus MBG 2-3 [16,17]. MBG: myocardial blush grade; OR: odds ratio; CI: confidence interval

Outcome	MBG 0-1 (n=80)	MBG 2-3 (n=120)	Statistical test	Test statistic	P-value	OR (95% CI)
Reperfusion success	55 (68.8%)	115 (95.8%)	χ² test	χ²=30.8	<0.001	8.85 (3.35-23.4)
Recurrent ischemia	11 (13.8%)	6 (5%)	χ² test	χ²=4.65	0.031	3.07 (1.05-8.95)
Malignant arrhythmias	9 (11.3%)	7 (5.8%)	χ² test	χ²=1.88	0.17	2.08 (0.73-5.93)
Acute heart failure	14 (17.5%)	9 (7.5%)	χ² test	χ²=4.89	0.027	2.63 (1.06-6.53)
Cardiogenic shock	6 (7.5%)	3 (2.5%)	Fisher's exact test	-	0.17	3.15 (0.73-13.5)
In-hospital mortality	7 (8.8%)	2 (1.7%)	Fisher's exact test	-	0.037	5.58 (1.09-28.4)

The results of the multivariate logistic regression analysis are presented in Table 4. MBG 0-1 continued to be an independent predictor of adverse in-hospital outcomes after accounting for prospective confounders (OR: 3.42; 95% CI: 1.48-7.91; p=0.004). Prolonged door-to-balloon time (>90 minutes) (OR: 2.65; 95% CI: 1.16-6.05; p=0.021) and anterior wall MI (OR: 2.28; 95% CI: 1.01-5.14; p=0.046) were also significant predictors. Age ≥60 years and diabetes mellitus demonstrated a trend toward an elevated risk, but this trend did not reach statistical significance.

**Table 4:** Multivariate logistic regression for predictors of in-hospital adverse outcomes Low MBG (0-1), prolonged door-to-balloon time >90 minutes, and anterior wall MI were independent predictors of adverse outcomes. Age ≥60 years and diabetes mellitus showed nonsignificant trends. MBG grading was performed per van't Hof et al. [17]. MBG: myocardial blush grade; MI: myocardial infarction; OR: odds ratio; CI: confidence interval

Predictor	Adjusted OR	95% CI	χ²	P-value
MBG 0-1 vs. 2-3	3.42	1.48-7.91	8.30	0.004
Age ≥60 years	2.01	0.91-4.47	3.02	0.08
Diabetes mellitus	1.87	0.81-4.30	2.18	0.14
Door-to-balloon >90 minutes	2.65	1.16-6.05	5.30	0.021
Anterior wall MI	2.28	1.01-5.14	3.98	0.046

**Figure 1:**
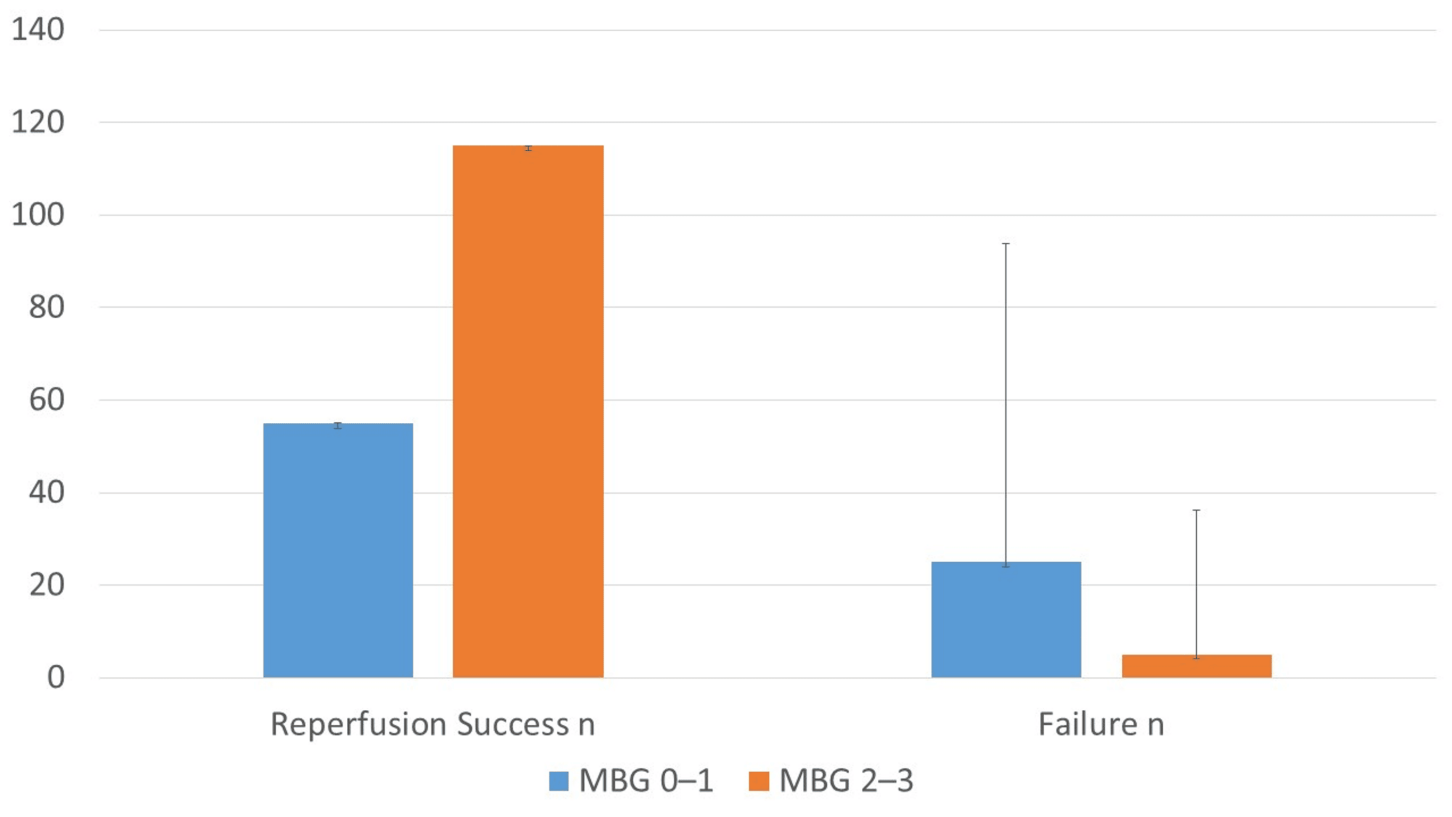
Reperfusion success across MBG groups Patients with MBG 2-3 achieved higher reperfusion rates than those with MBG 0-1 (p<0.001). MBG grading was performed per van't Hof et al. [17]; reperfusion success was defined as TIMI 3 flow plus MBG 2-3 [16,17]. MBG: myocardial blush grade; TIMI: Thrombolysis in Myocardial Infarction

Reperfusion success was markedly higher in patients with MBG 2-3 compared to those with MBG 0-1 (95.8% vs. 68.8%). The bar chart in Figure 1 demonstrates a clear gradient, highlighting the strong association between higher MBG and improved reperfusion success.

**Figure 2:**
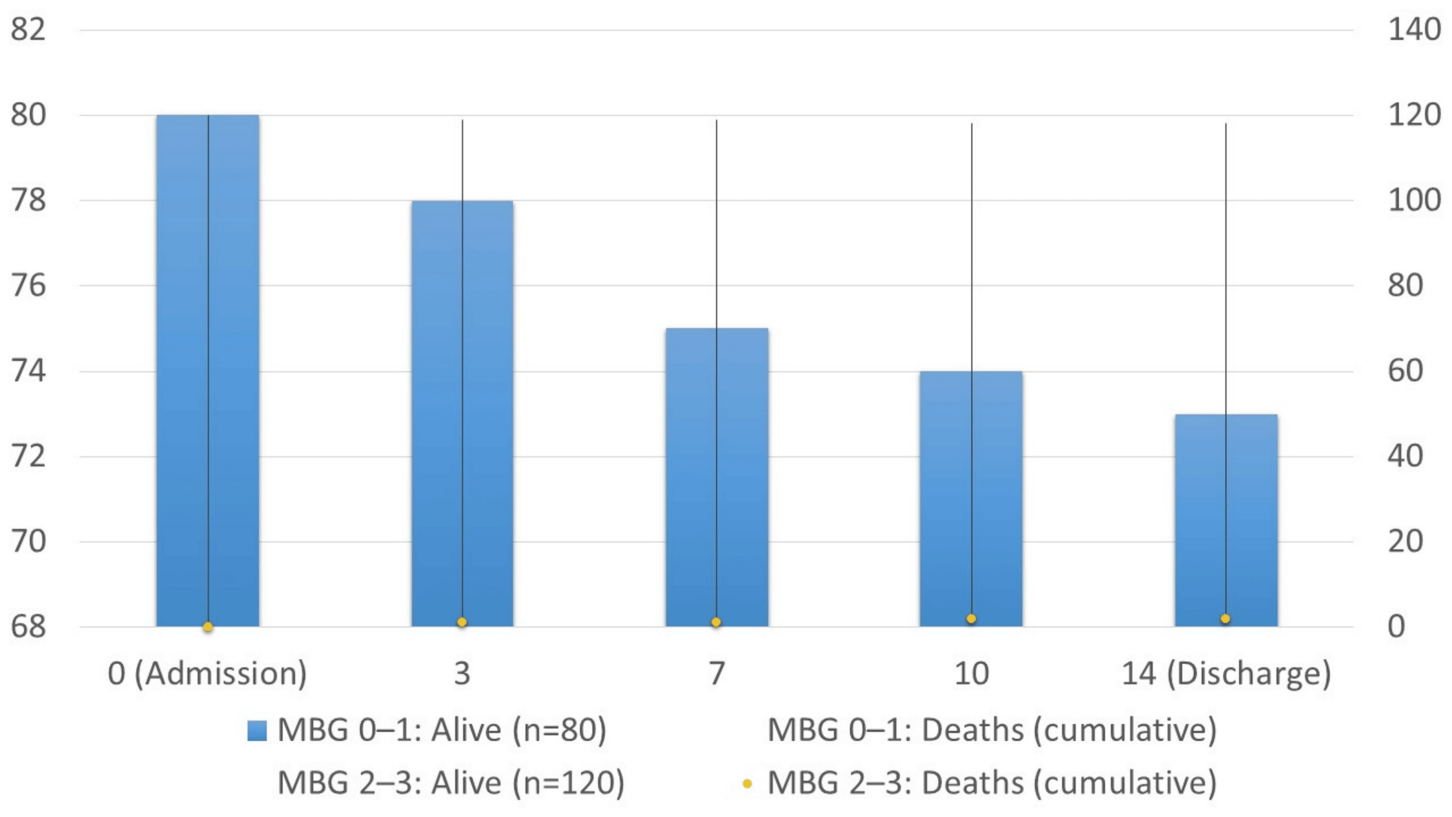
In-hospital mortality (up to 14 days) Patients with MBG 0-1 experienced significantly higher in-hospital mortality than those with MBG 2-3. Survival curves diverged within the first week and remained separated until discharge. MBG grading was performed per van't Hof et al. [17] and STEMI diagnosis per ESC/ACCF/AHA/WHF guidelines [14,15]. MBG: myocardial blush grade; ESC/ACCF/AHA/WHF: European Society of Cardiology/American College of Cardiology Foundation/American Heart Association/World Heart Federation; STEMI: ST-segment elevation myocardial infarction

The survival analysis in Figure 2 revealed that patients with MBG 0-1 experienced significantly higher in-hospital mortality than those with MBG 2-3 (8.8% vs. 1.7%). The survival curves began to diverge within the first week of hospitalization and remained separated until discharge, reflecting the prognostic value of MBG in predicting short-term outcomes.

**Figure 3:**
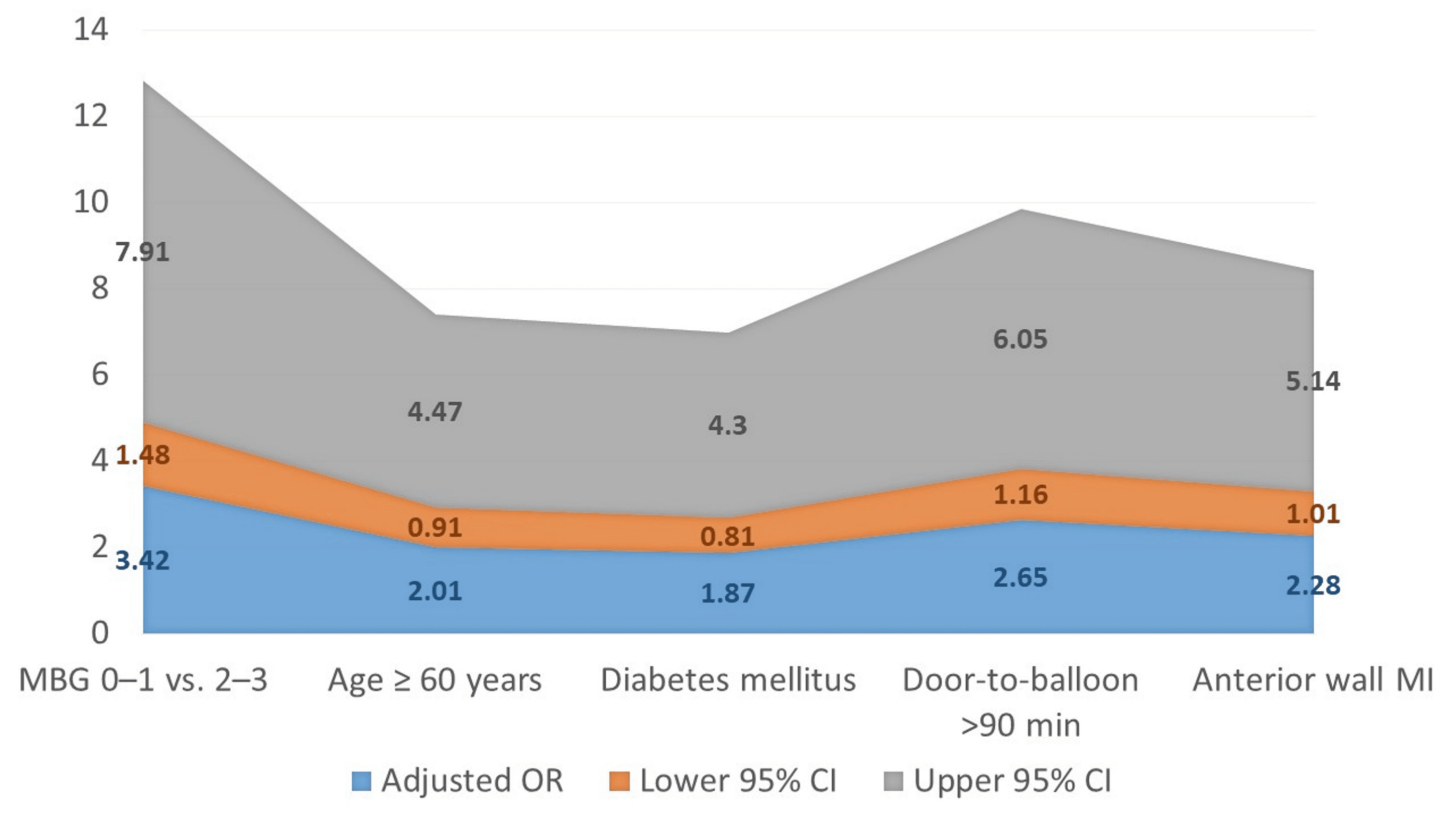
Predictors of in-hospital adverse outcomes Low MBG (0-1) was the strongest independent predictor, followed by prolonged door-to-balloon time and anterior wall MI. Age ≥60 years and diabetes mellitus showed nonsignificant trends. MBG grading was performed per van't Hof et al. [17] and TIMI flow grading per Chesebro et al. [16]. MBG: myocardial blush grade; OR: odds ratio; CI: confidence interval; MI: myocardial infarction; TIMI: Thrombolysis in Myocardial Infarction

Figure 3 illustrates the adjusted OR for predictors of in-hospital adverse outcomes. Low MBG (0-1) was the strongest independent predictor (OR: 3.42; 95% CI: 1.48-7.91), followed by prolonged door-to-balloon time (OR: 2.65; 95% CI: 1.16-6.05) and anterior wall MI (OR: 2.28; 95% CI: 1.01-5.14). Age ≥60 years and diabetes showed a trend toward increased risk but did not reach statistical significance.

## Discussion

This study demonstrated that MBG is a strong predictor of reperfusion success and in-hospital outcomes in STEMI patients treated with primary PCI at a tertiary care center in Pakistan. Patients with MBG 0-1 were older, had longer door-to-balloon times, and more frequently presented with anterior wall infarctions compared to those with MBG 2-3. Importantly, they achieved lower rates of reperfusion success and experienced higher rates of recurrent ischemia, acute heart failure, and in-hospital mortality. These associations persisted even after adjustment for confounding factors, establishing MBG as an independent prognostic marker.

Our findings are consistent with van 't Hof et al., who established MBG as a reliable angiographic marker of reperfusion and demonstrated its prognostic significance for clinical outcomes following primary PCI [17]. Brener et al. [1], in the INFUSE-AMI (Intracoronary Abciximab and Aspiration Thrombectomy in Patients With Large Anterior Myocardial Infarction) trial, further showed that patients with large anterior MI and impaired myocardial blush experienced significantly larger infarct sizes and higher mortality [1]. In the current cohort, the higher prevalence of anterior wall MI in the low MBG group likely contributed to their worse prognosis, which is in agreement with these prior reports.

The results also reinforce the critical importance of reperfusion time. Patients with door-to-balloon times greater than 90 minutes were at significantly higher risk of adverse events, consistent with data showing that myocardial salvage is highly time-dependent [16,18,19]. Notably, patients with MBG 0-1 had worse outcomes despite achieving post-PCI TIMI 3 flow, underscoring the superiority of MBG in capturing microvascular dysfunction and its incremental prognostic value over angiographic TIMI flow grade.

In-hospital mortality in the low MBG group (8.8%) was substantially higher compared with patients achieving MBG 2-3 (1.7%). These figures are comparable to mortality differences reported in prior observational studies. Raghavan et al. documented nearly a fourfold higher mortality in patients with MBG ≤1 compared to those with MBG ≥2 [18], while Yusuf et al. identified impaired myocardial blush as a strong predictor of left ventricular dysfunction and early death [19]. The consistency of our findings with these international studies reinforces the validity of MBG as a prognostic tool in STEMI.

Multivariate analysis in this study further identified MBG 0-1, prolonged door-to-balloon time, and anterior wall MI as independent predictors of in-hospital adverse outcomes. Although diabetes and advanced age showed trends toward increased risk, they did not reach statistical significance in this cohort, possibly reflecting improvements in medical management and limited sample size. Similar observations have been reported in contemporary registries where aggressive secondary prevention therapies mitigate the impact of traditional risk factors [20]. Notably, impaired myocardial blush, particularly in patients with anterior wall infarctions, has been consistently associated with larger infarct size and higher short-term mortality, highlighting its value as a robust prognostic marker [21].

The MBG provides incremental prognostic information beyond TIMI flow, as it reflects both epicardial patency and microvascular integrity. Incorporating MBG into routine angiographic assessment may enhance early risk stratification and help clinicians identify high-risk patients who might benefit from closer monitoring or adjunctive therapies. In the context of Pakistan, where STEMI continues to impose a major health burden and timely access to PCI is often limited, adopting simple and cost-effective prognostic markers such as MBG could play an important role in improving patient outcomes.

Strengths and limitations

This study has several notable strengths. It employed a prospective observational design with an adequately powered sample size determined through formal statistical estimation, enhancing the robustness of its findings. The standardized angiographic evaluation of MBG was conducted by two independent, experienced interventional cardiologists who were blinded to patient outcomes, with strong inter-observer agreement (κ=0.84) confirming internal reliability. Procedural uniformity was maintained through pre-specified institutional protocols and checklists, which improved consistency and facilitated reproducibility across cases. The use of multivariate logistic regression further minimized the influence of confounding factors, ensuring a more accurate estimation of independent predictors. Additionally, the study was carried out in a high-volume tertiary cardiac care center, providing data that closely reflect real-world clinical practice in a developing healthcare setting.

However, certain limitations must be acknowledged. First, this was a single-center study, which may restrict the generalizability of results to different populations or institutional protocols. Second, the follow-up period was limited to in-hospital outcomes, precluding evaluation of long-term prognostic implications of MBG and its association with post-discharge events. Third, despite structured observer training and calibration, MBG assessment remains partly subjective, and the absence of angiographic core-lab validation may introduce minor variability. Although inter-observer reliability was periodically reassessed to maintain consistency, minor interpretive drift over time cannot be entirely excluded. Lastly, residual confounding from unmeasured clinical or procedural variables (e.g., microvascular dysfunction, ischemia duration, or periprocedural medication differences) could not be fully accounted for and may have influenced reperfusion outcomes.

## Conclusions

This study suggests that MBG is a strong prognostic marker associated with better reperfusion success, lower in-hospital mortality, and fewer adverse cardiovascular events among STEMI patients undergoing primary PCI. While these findings indicate a robust association rather than a causal relationship, they highlight the clinical relevance of MBG as a simple, cost-effective, and reproducible angiographic parameter for early risk stratification and outcome prediction. Given the single-center nature of this study, the results primarily reflect the characteristics of the population treated in a tertiary cardiac care setting in Pakistan and may not be generalizable to all healthcare environments. Future multicenter investigations with larger and more diverse cohorts, as well as longer follow-up durations, are warranted to validate these associations and to better define the long-term prognostic implications of MBG following primary PCI.

## References

[1] Brener SJ, Maehara A, Dizon JM (2013). Relationship between myocardial reperfusion, infarct size, and mortality: the INFUSE-AMI (Intracoronary Abciximab and Aspiration Thrombectomy in Patients With Large Anterior Myocardial Infarction) trial. JACC Cardiovasc Interv.

[2] Takagi K, Tanaka A, Yoshioka N (2021). In-hospital mortality among consecutive patients with ST-elevation myocardial infarction in modern primary percutaneous intervention era: insights from 15-year data of single-center hospital-based registry. PLoS One.

[3] Akhtar A, Saleemi MS, Zarlish QM, Arshad MB, Hashmi KA, Ghafoor H (2023). Experience and outcomes of primary percutaneous coronary intervention in a tertiary care hospital in South Punjab, Pakistan. Cureus.

[4] Guerchicoff A, Brener SJ, Maehara A (2014). Impact of delay to reperfusion on reperfusion success, infarct size, and clinical outcomes in patients with ST-segment elevation myocardial infarction: the INFUSE-AMI trial (INFUSE-Anterior Myocardial Infarction). JACC Cardiovasc Interv.

[5] Scholz KH, Maier SK, Maier LS (2018). Impact of treatment delay on mortality in ST-segment elevation myocardial infarction (STEMI) patients presenting with and without haemodynamic instability: results from the German prospective, multicentre FITT-STEMI trial. Eur Heart J.

[6] Kumar R, Khan KA, Rahooja K (2023). Outcomes of ST-segment elevation myocardial infarction in a cohort of cardiogenic shock patients undergoing primary percutaneous coronary intervention. Pak Heart J.

[7] Ali N, Khan MA, Shinwari MI, Ullah I (2024). In-hospital outcomes of patients with anterior wall myocardial infarction and right bundle branch block in the primary PCI era: impact and prognostic factors. Pak Heart J.

[8] Shahzad K, Ali J, Ahmad A, Usman A, Rashdi A, Shafiq F (2020). Outcome of primary percutaneous coronary intervention in patients with acute ST-segment elevation myocardial infarction arriving in Army Cardiac Center Lahore. Pak Armed Forces Med J.

[9] Mehta S, Granger CB, Henry TD (2017). Reducing system delays in treatment of ST elevation myocardial infarction and confronting the challenges of late presentation in low and middle-income countries. Indian Heart J.

[10] Kodeboina M, Piayda K, Jenniskens I (2023). Challenges and burdens in the coronary artery disease care pathway for patients undergoing percutaneous coronary intervention: a contemporary narrative review. Int J Environ Res Public Health.

[11] Rathore SS, Curtis JP, Chen J, Wang Y, Nallamothu BK, Epstein AJ, Krumholz HM (2009). Association of door-to-balloon time and mortality in patients admitted to hospital with ST elevation myocardial infarction: national cohort study. BMJ.

[12] Lwanga SK, Lemeshow S (1991). Sample Size Determination in Health Studies: A Practical Manual. https://tbrieder.org/publications/books_english/lemeshow_samplesize.pdf.

[13] Che W, Jin Y, Chang S, Sun Y, Hou A, Wang C (2025). Prediction of myocardial ischemia-reperfusion injury post-PCI: role of sST2 levels in STEMI patients. BMC Cardiovasc Disord.

[14] Thygesen K, Alpert JS, Jaffe AS (2012). Third universal definition of myocardial infarction. Eur Heart J.

[15] Thygesen K, Alpert JS, Jaffe AS (2018). Fourth universal definition of myocardial infarction. Circulation.

[16] Chesebro JH, Knatterud G, Roberts R (1987). Thrombolysis in myocardial infarction (TIMI) trial, phase I: a comparison between intravenous tissue plasminogen activator and intravenous streptokinase. Clinical findings through hospital discharge. Circulation.

[17] van't Hof AW, Liem A, Suryapranata H, Hoorntje JC, de Boer MJ, Zijlstra F (1998). Angiographic assessment of myocardial reperfusion in patients treated with primary angioplasty for acute myocardial infarction: myocardial blush grade. Zwolle Myocardial Infarction Study Group. Circulation.

[18] Raghavan S, Vassy JL, Ho YL (2019). Diabetes mellitus-related all-cause and cardiovascular mortality in a national cohort of adults. J Am Heart Assoc.

[19] Yusuf J, Das D, Mukhopadhyay S, Tyagi S (2018). Correlation of QRS duration with myocardial blush grade as a marker of myocardial reperfusion in primary percutaneous coronary intervention. Indian Heart J.

[20] Liu Y, Geng N (2025). Study on secondary prevention and the impact of risk factor control on myocardial infarction events in young patients with coronary heart disease. Medicine (Baltimore).

[21] Tuka V, Holub J, Bělohlávek J (2022). Secondary prevention after myocardial infarction: what to do and where to do It. Rev Cardiovasc Med.

